# Pathological Accuracy in Prostate Cancer: Single-Center Outcomes of 3 Different Magnetic Resonance Imaging-Targeted Biopsy Techniques and Random Systematic Biopsy

**DOI:** 10.5152/tud.2022.22165

**Published:** 2022-09-01

**Authors:** Mert Kılıç, Ömer Acar, Metin Vural, Bülent Çolakoğlu, Barbaros Erhan Çil, Ersin Köseoğlu, Dilek Ertoy Baydar, Abdullah Erdem Canda, Yakup Kordan, Mevlana Derya Balbay, Tarık Esen

**Affiliations:** 1 Department of Urology, VKF American Hospital, İstanbul, Turkey; 2 Department of Urology, Koç University Faculty of Medicine, İstanbul, Turkey; 3 Department of Radiology, VKF American Hospital, İstanbul, Turkey; 4 Department of Radiology, Koç University Faculty of Medicine, İstanbul, Turkey; 5 Department of Pathology, Koç University Faculty of Medicine, İstanbul, Turkey

**Keywords:** Prostate cancer, Gleason score, prostatectomy, biopsy, Multiparametric MRI

## Abstract

**Objective:**

The aim of this study is to compare systematic, cognitive fusion, in-bore, and software fusion prostate biopsies regarding rates of and risk factors for pathological upgrading.

**Material and methods:**

Charts of 291 patients with systematic biopsy (n = 105), magnetic resonance imaging-targeted cognitive fusion (n = 58), in-bore (n = 68), and software fusion biopsy (n = 60), and who subsequently underwent radical prostatectomy were retrospectively evaluated. The degree of similarity between the grade groups reported in the biopsy and radical prostatectomy pathology results was recorded. Analyses of the associated factors for concordance and discordance were performed with univariate and multivariate methods.

**Results:**

The concordance rates were as follows: systematic biopsy = 42.8%, cognitive fusion-targeted biopsy = 50%, in-bore fusion-targeted biopsy = 61.8, and software fusion biopsy = 58.4%. The upgrade rate of systematic biopsy (46.6%) was higher than cognitive fusion-targeted biopsy (27.6%), in-bore fusion-targeted biopsy (26.4%), and software fusion-targeted biopsy (18.3%). The number of positive cores was significantly associated with grade group concordance for the systematic biopsy group (*P* = .040). Within the cognitive fusion-targeted biopsy cohort, number of positive cores was the only parameter that exhibited a significant association with grade group concordance in multivariate analysis (*P* = .044). Considering the in-bore fusion-targeted biopsy group, maximum tumor length was statistically significant (*P* = .021). In the software fusion-targeted biopsy group, low prostate volume was found to be the only significant predictor for grade group accordance (*P* = .021).

**Conclusion:**

Magnetic resonance imaging-targeted biopsy techniques showed higher concordance and lower upgrade rates compared to systematic biopsy. For systematic biopsy and cognitive fusion-targeted biopsy, the number of positive cores was associated with grade group concordance, while maximum tumor length in in-bore fusion-targeted biopsy and low prostate volume for in-bore fusion-targeted biopsy were associated with grade group concordance. Among the MRI-targeted biopsy methods, in-bore fusion-targeted biopsy and software fusion-targeted biopsy were more accurate than cognitive fusion-targeted biopsy in terms of grade group.

**Main Points**

- Magnetic resonance imaging (MRI)-targeted biopsy techniques showed higher concordance and lower upgrade rates compared to systematic biopsy.
- Among the MRI-targeted biopsy methods, in-bore fusion-targeted biopsy and software fusion-targeted biopsy were more accurate in terms of grade group when compared to cognitive fusion-targeted biopsy.
- The number of positive cores was associated with grade group concordance for systematic biopsy and cognitive fusion-targeted biopsy technique.
- The maximum tumor length was the only variable demonstrating a similar association for in-bore fusion-targeted biopsy.
- Contrary to the random systematic biopsy-period studies, low prostate volume was associated with grade group concordancy for software fusion-targeted biopsy.

## Introduction

Tumor grade is one of the most important pathological parameters that needs to be considered while making treatment decisions in prostate cancer (PCa). Underestimation of the Gleason grade is a prevalent problem, reported being as high as 43% in contemporary series.^1-3^

Magnetic resonance imaging (MRI)-targeted biopsy (MRTB) of the prostate is gaining popularity due to its higher diagnostic yield for the detection of clinically significant PCa and superior ISUP grade group (GG) concordance rate between biopsy and radical prostatectomy (RP) specimens when compared to random systematic biopsy (SB).^4^ Selective sampling of suspicious lesions with the guidance of MRI may result in better concordance with the RP pathology owing to a higher percentage of cancer per core. However, even MRTB techniques have not reached excellent GG prediction rates yet.

To date, several studies have reported GG concordance rates with SB and different MRTB techniques with substantial heterogeneity in the outcomes.^5^ Some of these studies presented SB and MRTB biopsy outcomes concurrently, while others had different cohorts. However, the literature is devoid of a single-center study analyzing the outcomes of all three MRTB techniques (cognitive fusion (CF-TB), in-bore (IB-TB), and software fusion (SF-TB)) in terms of GG concordance between biopsy and RP specimens in a standardized manner.

In this study, we evaluated the grading performance of systematic and MRTB techniques and the factors predicting accurate grading in patients who were treated with RP.

## Materials and Methods

Between 2014 and 2020, data of 291 patients who were diagnosed with PCa using SB (10-14 cores) and MRTB and subsequently underwent RP were retrospectively evaluated. Patients who underwent MRTB due to a <PI-RADS-4 lesion, patients with prior prostate surgery, and patients who received androgen deprivation treatment, or are on active surveillance were excluded. Patients who underwent a biopsy outside our center were included in the study as well. Age, digital rectal examination (DRE), prostate-specific antigen (PSA [ng/mL]), PSA density, prostate volume (mL) defined by MRI, previous biopsy status, PI-RADS score, index lesion length, the number of total biopsy core, the number of tumor positive cores, and the maximum tumor length among all cores were recorded for all patients.

Of the remaining 291 patients included in the study, 105 (36.1%) underwent SB (Group 1), 58 (19.9%) underwent CF-TB (Group 2), 68 (23.3%) underwent IB-TB (Group 3), and 60 (20.7%) underwent SF-TB (Group 4).

All SB procedures were carried out with a standard transrectal biopsy technique, including 10 to 12 cores. About half of the patients with SB consisted of patients who already had biopsy results from external centers. The remaining patients did not have any suspicious lesions on their MRI (<PI-RADS 4) but had elevated PSA or positive DRE, and were considered for SB. The patients from external centers who had SB without MRI underwent preoperative MRI in our center. Thus, the SB group included not only patients with <PI-RADS 4 lesions but also those with PI-RADS 4 and 5 lesions.

Magnetic resonance imaging -targeted prostate biopsy was selected according to the patient’s and surgeon’s preference. In-bore biopsy was performed on the same 3T MRI scanner by a radiologist (MV) who had 12 years of experience in urogenital radiology and interventions. In-bore MRI-guided biopsy was recommended for the patients with targetable single index lesions with special emphasis on the anterior location.

Cognitive fusion biopsies were performed by one urologist (DB) or interventional radiologist (BÇ). The images were analyzed and index lesions were sampled, ranging between two to and 5 core biopsies per lesion. After the targeted lesion was obtained, patients underwent an SB including 10 to 12 cores.

Software fusion biopsies were performed by one urologist (YK) and one interventional radiologist (BÇ). Biopsies of the target lesions were carried out using UroNav Fusion Biopsy System (Philips-Invivo, Gainesville, FL) followed by 10 to 12-core systematic transrectal ultrasound-guided biopsies.

All pathological materials were evaluated by a single very experienced uropathologist (DRB). The ISUP GG in prostate biopsy and RP specimens were recorded. The concordance, upgrade, and downgrade rates and associated clinicopathological factors were analyzed. The study was conducted following institutional review board approval from Koç University (2022. 228.I RB1.0 83-17 .06.2 022) and verbal informed consent was obtained from all participants in the study.

### Statistical Analysis

Continuous variables were reported using mean, median, and range. Categorical variables were reported as percentages. Student’s t-test or the Mann-Whitney U test was used for the parametric and nonparametric variables. A chi-square test was performed for the categorical variables. The Kruskal-Wallis test was employed for comparing the variables of the 4 different biopsy techniques. The analysis of the associated factors for concordance and upgrade was performed using univariate (chi square and Student’s t-test) and multivariate (logistic regression) methods. Analyses were performed using the IBM SPSS Statistics for Windows, Version 26.0 (IBM SPSS Corp.; Armonk, NY, USA) *P* value of <.05 was considered significant.

## Results

**Figure 1.**
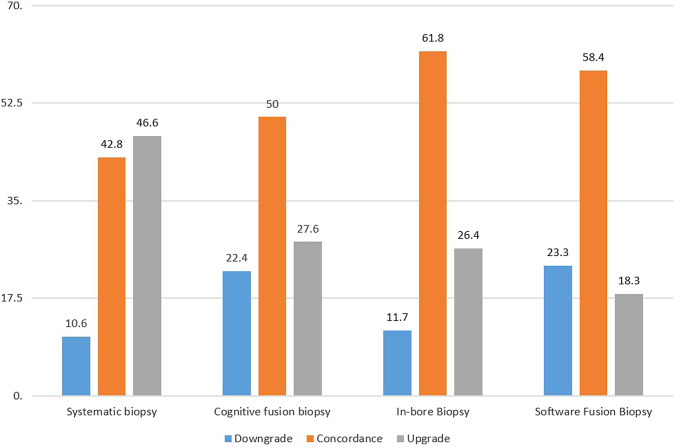
The downgrade, concordance, and upgrade rates of each biopsy technique.

Patient demographics are shown in Table 1. The PI-RADS 4 and PI-RADS 5 lesion distribution was similar among the MRTB biopsy techniques. In Group 1, 54 (51.4%) patients underwent multiparametric MRI and 76% of them had PI-RADS 4 or 5 lesions. The PI-RADS and Gleason GG distribution regarding the biopsy and RP findings are shown in Table 2. The absolute concordance of GG between biopsy and RP for each biopsy technique was 42.8% in Group 1, 50.0% in Group 2, 61.8% in Group 3, and 58.4% in Group 4 (Figure 1). The upgrade rate was higher (46.6%) in Group 1 than in Group 2 (27.6%), Group 3 (26.4%), and Group 4 (18.3%). The downgrade was higher in Group 2 (22.4%) and Group 4 (23.3%) when compared to Group 1 (10.6%) and Group 3 (11.7%).

For the SB group, the number of positive biopsy cores was the only parameter that was significantly higher in patients showing GG concordance (5.3 ± 3.3 vs. 4.0 ± 2.6, *P* = .040) (Table 3).

In the univariate analysis of CF-TB, prostate-specific antigen density (PSAD) (*P* = .037), the maximum tumor length of core (*P* = .038), and the number of positive cores (*P *= .001) were significantly higher in patients in concordance (Table 4). In multivariate analysis, the number of positive cores was the only parameter that was significantly associated with concordance (OR = 0.688, 95% CI: 0.477-0.990, *P* = .044).

Univariate analysis of the predictors for concordance and upgrade for IB-TB is shown in Table 5. Only maximum tumor length was significantly associated with concordance (9.2 ± 3.9 mm vs. 6.9 ± 3.6 mm, *P *= .021). For SF-TB, the prostate volume was the only parameter that was found to be significant between concordance and upgrade groups (40 [13-120] vs. 52.5 [28-131], *P* = .021) (Table 6).

As for the patients who had biopsy GG 1 or 2, the concordance rates were 77%, 83.9%, 86%, and 91% for the SB, CF-TB, IB-TB, and SF-TB biopsy groups, respectively (*P* = .111). On the other hand, the rate of upgrade to GG 3≤ was 23%, 16.1%, 14%, and 9%, respectively.

## Discussion

Many factors may lead to discordance between biopsy and RP grading. Different discordance rates ranging between 28% and 76% were reported so far and have been attributed to pathological misinterpretation,^6,7^ sampling error,^2,8-10^ and baseline demographics^11^ Therefore, several studies have focused on predicting factors to reach higher biopsy accuracy.

Bullock et al^11^ reported the grading accuracy of 17 598 patients from the British Association of Urological Surgeons Radical Prostatectomy Registry database of prospectively entered cases. The exact type of biopsy technique was not recorded; nevertheless, the authors stated that Transrectal ultrasound (TRUS)-guided biopsy utilizing an extended sampling approach was the common approach during their study period. The concordance, upgrade, and downgrade rates were 58.9%, 25.5%, and 15.6%, respectively. This study had a lower number of low-risk patients compared to our study (10% vs. 14.7%), and the rate of upgrading from low-risk disease (55.7%) was lower compared to our result (82.9%). These different rates show the substantial role of baseline demographics such as preoperative Gleason grade, PSA, and stage on GG concordance.

In the current study, the rate of GG concordance between biopsy and RP for each biopsy technique was 42.8% for SB, 50.0% for CF-TB, 61.8% for IB-TB, and 58.4% for SF-TB. The upgrade rate was higher for SB (47.2%) than CF-TB (27.6%), IB-TB (26.4%), and SF-TB (18.3%). In a systematic review and meta-analysis, Goel et al^4^ reported a 23.3% upgrade rate for targeted biopsy (TB) irrespective of the MRTB technique, whereas the upgrade rate was 42.7% for SB. No significant difference was detected in terms of pathologic downgrading between MRTB and SB.

In a study evaluating the value of combining SB and SF-TB, Ploussard et al^12^ reported an increase in concordance rates from 35.6% to 45.2% between SB and SF-TB, and from 45.2% to 51.7% between SF-TB and SF-TB plus SB. However, the upgrade rate decreased by 22% when SB was added to TB.

In another study, Ahdoot et al^13^ reported the outcomes of 404 patients who underwent combined biopsy. The rate of pathological upgrade for SB, TB, and the combination of SB and TB was 41.6%, 30.9%, and 14.4%, respectively. The rate of upgrading from GG 1 or 2 to GG ≥ 3 was 16.8%, 8.7%, and 3.5%, respectively. In our study, the upgrade rate to GG ≥ 3 was 23% for SB, and slightly lower for MRTB (CF-TB = 14%, IB-TB = 16.1%, SF-TB = 9%). In other words, concordance rates within GG 1 or 2 were 77%, 83.9%, 86%, and 91% for the SB, CF-TB, IB-TB, and SF-TB biopsy groups, respectively (*P* = .111). The upgrade rate from GG ≤ 2 was reported to range between 24.8% and 45.8 % in the literature.^12,14-16^

In the current study, various variables were found to be significant in predicting the GG accurately. In our SB group, the number of positive biopsy cores was the only factor significantly associated with GG concordance (5.3 ± 3.3 vs. 4 ± 2.6, *P* = .040). Similarly, it was the only parameter that exhibited significant association with GG concordance in the CB group in the multivariate analysis. As for IB-TB, maximum tumor length was significantly associated with concordance (9.2 ± 3.9 mm vs. 6.9 ± 3.6 mm, *P* = .021). Finally, in SF-TB, prostate volume was the only variable that was found to be significant in predicting GG concordance. Many studies have demonstrated different predictors of GG concordance so far; some of them showed that smaller prostate was a predictor of pathological upgrading.^17-20^ However, it is noteworthy that these studies belong to the period before MRTB. Conversely, in our study, large prostate volume predicted the upgrade in SF-TB. In fact, the prostate volume of those who were upgraded in other MRTBs was higher, as in SF-TB, although there was no statistically significant difference. On the other hand, prostate volume was similar among patients who had an upgrade and concordant in SB biopsy. The association between large prostate volume and upgrade has been supported by limited post-MRTB studies so far.^21^

Higher PSA was also found to be a predictor of GG upgrading.^11^ Ploussard et al^12^ reported that patients with a PSAD < 0.20 ng/mL/g benefited from significantly greater grading concordance with a combined (systematic and targeted) biopsy strategy.^12^ Maruyama et al indicated PI-RADS score as a predictor of GG upgrading in addition to PSAD for patients with GG1 disease.^22^

The number of biopsy cores was also linked to upgrading not only for SB but also for MRTB biopsies.^23^ The optimal number of cores to be obtained by TB for an accurate diagnosis is still controversial and reported to range between 1 and 6 cores per index lesion.^24,25^ In our study, the number of cores was almost 4 per index lesion in all MRTB methods and no statistically significant effect was found in upgrading. Muthigi et al^21^ reviewed patients who underwent Multiparametric-magnetic resonance imaging (mpMRI) followed by SF-TB and SB from a prospectively managed database.^21^ For SB, in addition to higher prostate volume and a lower PSA, a lower number of target cores was suggested as an independent predictor for upgrading. Intratumoral Gleason score heterogeneity was also implicated as another cause of upgrading in SB over SF-TB. Utilizing MRTB provides a higher percentage of tumor per core.^26,27^ Furthermore, IB-TB biopsy provides real-time MRI guidance during tissue sampling and allows for higher accuracy and better diagnostic yield, supporting its potential superiority over other MRTBs. Osses et al^28^ demonstrated that PCa detection rate in relatively smaller index lesions (0-1.5 mL) was significantly higher in IB-TB when compared to CF-TB (69% vs. 39%, respectively). In addition, some studies have shown that CF-TB is not inferior to SF-TB, but index lesion may be better focused with a software-based approach. Eventually, CF-TB can give better results in the presence of non-small index lesions as previous studies suggested.^29^ Another study suggested that IB-TB was associated with a higher amount of malignant tissue within the biopsy core compared to SF-TB.^30^ In our study, tumor length was found to be the only predictor of GG concordance in the IB-TB group. So far, several studies showed that biopsy tumor length predicts pathologic upgrade.^31,32^ The risk of upgrading was found to be increased two-fold in patients with cancer involving >5 mm of the biopsy core.^31^ Literature is scarce in studies comparing IB-TB with other biopsy techniques in terms of GG accuracy. In one study, IB-TB was compared with SF-TB, and upgrade rates were reported to be 17% versus 27% for IB-TB and SF-TB, respectively (*P* = .55).^33^ In our study, the upgrade rate of SF-TB (18.3%) was slightly less than IB-TB (26.4%) and CF-TB (27.6%). On the other hand, IB-TB does not allow for concomitant SB due to time limitation.^33^ Consequently, some of the significant tumor foci might be left unsampled. It was reported that up to 10% of significant cancer can be missed by an index lesion-only approach.^34,35^ Therefore, that limitation of IB-TB might be restrictive to make a more significant difference in GG accordance.

In a study investigating the effect of previous biopsy status on the results of MRTB and SB, PCa rates were found to be higher in biopsy-naive patients compared to patients with one previous biopsy (biopsy naïve; SB = 67.4%, TB + SB = 71.6%, patients with one previous biopsy; SB = 43.6%, TB + SB = 50.9%, *P* < .01.).^36^ Moreover, in the meta-analysis of Goel et al mentioned before, in biopsy naïve subgroup analysis, upgrade rate of SB was found to be 2.47 times higher than in TB. In our study, a significant majority (about 90%) of all biopsy methods consisted of biopsy-naïve patients, and the upgrade rates were in line with Goel’s study.^4^

To our knowledge, this is the first single-center study that compares the GG concordance of SB and 3 different MRTB methods. One of the main strengths of this study is the well-established standardized protocols for each MRTB technique. The other one is the performance of the pathological evaluations by two uropathologists from the same institution with high experience. However, our study had some limitations. This is a retrospective, non-randomized study. Each technique was performed by different surgeons, with some of our patients in the SB group biopsied by external centers. The number of cases for each biopsy technique was limited and might have affected the statistics. Unlike other MRTB approaches, the lack of SB in IB-TB might influence cancer detection rate and histologic grade accuracy. It is noteworthy to emphasize that these results should be confirmed by perineal biopsy, which is now recommended as the first choice by EAU guidelines.^37^

In conclusion, all MRTB techniques showed higher concordance and lower upgrade rates compared to SB. For systematic and cognitive fusion biopsy, the number of positive cores was associated with ISUP GG concordance between biopsy and RP specimens. On the other hand, for IB-TB, the maximum tumor length was the only variable demonstrating a significant association with GG concordance, and contrary to the SB period studies, for SF-TB, low prostate volume was associated with GG concordancy.

Multiparametric prostate MRI has a substantial role in identifying the dominant lesion. Among the MR-TB methods, IB-TB and SF-TB were more accurate in terms of GG when compared to CF-TB.

## Figures and Tables

**Table 1.** Summary of the Clinical Characteristics of the Study Cohort

	Group 1 Systematic Biopsy (n = 105)	Group 2 Cognitive Fusion Biopsy (n = 58)	Group 3 In-Bore Biopsy (n = 68)	Group 4 Software Fusion Biopsy (n = 60)	*P*
Age (years)	62.2 ± 7.2	64.1 ± 7.3	63.2 ± 6.1	64.4 ± 10.0	.232
Abnormal DRE	57 (54%)	32 (55%)	24 (35%)	23 (38.3%)	.020
Median (range) PSA value (ng/mL)	8 (3-46)	6.5 (2.4-60)	5.45 (2.1-26)	6.11 (1.1-1.40)	<.001
Mean prostate volume (ml)	48.9 ± 22.3	51.4 ± 31.8	47.3 ± 21.5	52.5 ± 32.4	.920
PSAD	0.23 ± 0.22	0.25 ± 0.40	0.15 ± 0.09	0.17 ± 0.13	.011
Biopsy naïve (n)	92 (87.7%)	54 (93.1%)	62 (91.2%)	56 (93.3%)	.590
Maximum tumor length per core (mm)	7.9 ± 4.8	12.3 ± 6.2	8.8 ± 4.0	6.9 ± 3.8	<.01
Total number of cores (n)	12.7 ± 2.7	14.3 ± 1.8	4.1 ± 1.3	16.9 ± 2.3	<.001
Number of cores per index lesion (n)	–	4.3 ± 1.6	4.1 ± 1.3	4.2 ± 1.5	.151
Number of positive cores (%)	4.5 ± 3.0 (36%)	5.6 ± 3.3 (39%)	3.2 ± 1.1 (78%)	6.9 ± 3.9 (40.8%)	<.001
Index lesion diameter (mm)	14.7 ± 7.8	13.6 ± 6.1	12.8 ± 6. 8	15.3 ± 9.7	.425

DRE, digital rectal examination; PSA, prostate-specific antigen; PSAD, prostate-specific antigen density.

**Table 2.** PI-RADS Score and ISUD GG Distribution for Each Biopsy Technique

	Group 1 Systematic Biopsy (n = 105)	Group 2 Cognitive Fusion Biopsy (n = 58)	Group 3 In-Bore Biopsy (n = 68)	Group 4 Software Fusion (n = 60)
PI-RADS score				
1	3 (5%)	–	–	–
2	2 (4%)	–	–	–
3	8 (15%)	–	–	–
4	21 (38%)	36 (62.1%)	44 (64.7%)	36 (60)
5	20 (37%)	22 (37.9%)	24 (35.3%)	24 (40)
Bx GG				
1	38 (36.2%)	9 (16.1%)	12 (17.6%)	13 (21.7%)
2	36 (34.3%)	21 (35.7%)	31 (45.6%)	20 (33.3%)
3	17 (16.2%)	8 (14.3%)	13 (19.1%)	17 (28.4%)
4	8 (7.6%)	10 (16%)	9 (13.2%)	5 (8.3%)
5	6 (5.7%)	10 (17.9%)	3 (4.4%)	5 (8.3%)
RP GG				
1	6 (5.7%)	2 (3.4%)	2 (2.9%)	6 (10%)
2	56 (53.3%)	31 (53.4%)	38 (55.9%)	34 (56.6%)
3	25 (23.8%)	12 (20.1%)	17 (25%)	15 (25%)
4	6 (5.7%)	1 (1.7%)	5 (7.4%)	1 (1.7%)
5	12 (11.4%)	11 (19%)	6 (8.8%)	4 (6.7%)

Bx, biopsy; RP, radical prostatectomy.

**Table 3.** Univariate Analysis of Possible Predictors for Concordance and Upgrade in the SB Method

	Concordance (n = 45 [42.8%])	Upgrade (n = 49 [46.6%])	*P*
Age (years)	63.6	60.8	.066
Abnormal DRE	28 (62%)	24 (49%)	.188
PSA (median) ng/mL	8.7 (3.7-46.0)	7.3 (3.0-30.0)	.738
PSAD (median)	0.17 (0.06-1.00)	0.16 (0.05-0.70)	.711
Prostate volume (median) mL	45 (22-159)	46.5 (18-110)	.820
Maximum tumor length of core (mm)	8.0 ± 4.0	7.8 ± 5.8	.466
Number of positive cores	5.3 ± 3.3	4.0 ± 2.6	.040

DRE, digital rectal examination; PSA, prostate-specific antigen; PSAD, prostate-specific antigen density; SB, systematic biopsy.

**Table 4.** Univariate and Multivariate Analysis of Possible Predictors for Concordance and Upgrade in Cognitive-Fusion Biopsy

	Univariate			Multivariate	
	Concordance (n = 29)	Upgrade (n = 16)	*P*	OR (95% CI)	*P*
Age (years)	64.0 ± 7.1	62.9 ± 8.5	.830		
Abnormal DRE, n (%)	12 (41.3%)	9 (56.2%)	.368		
PSA (median) ng/mL	6.5 (4.0-21.5)	5.6 (2.5-19.1)	.643		
PSAD (median)	0.21 (0.04-2.6)	0.11 (0.03-0.4)	.037	0.002 (0-17.435)	.179
Prostate volume (median) ml	37.0 (19.0-120.0)	52.5 (30.0-172.0)	.108		
Max tumor length of core (mm)	13.3 ± 5.1	9.8 ± 8.2	.038	0.962 (0.856-1.081)	.517
Number of cores per index lesion	4.3 ± 1.3	4.3 ± 1.4	.343		
Number of positive cores	6.3 ± 3.5	3.0 ± 1.9	.001	0.688 (0.477-0.990)	.044
Index lesion diameter (mm)	14.9 ± 6.8	12 ± 5.0	.270		

DRE, digital rectal examination; PSA, prostate-specific antigen; PSAD, prostate-specific antigen density; OR, odds ratio.

**Table 5.** Univariate Analysis of Possible Predictors for Concordance and Upgrade in IB-TB

	Concordance (n = 42)	Upgrade (n = 18)	*P*
Age	64.0 ± 5.1	62.3 ± 7.7	.663
Abnormal DRE	18 (42.8%)	7 (38.9%)	.822
Median PSA	5.42	5.40	.545
Median PSAD	0.12	0.10	.27
Median prostate volume (mL)	45	49	.890
Max tumor length of core (mm)	9.2 ± 3.9	6.9 ± 3.6	.021
Number of cores per index lesion	4.2 ± 1.4	4.1 ± 1.4	.490
Number of positive cores	3.3 ± 1.1	2.9 ± 0.9	.276
Index lesion diameter (mm)	12.1 ± 5.8	14.8 ± 9.0	.360

DRE, digital rectal examination; PSA, prostate-specific antigen; PSAD, prostate-specific antigen density; IB-TB, in-bore fusion-targeted biopsy.

**Table 6.** Univariate Analysis of the Possible Predictors for Concordance and Upgrade in Software Fusion Biopsy

	Concordance (n = 35)	Upgrade (n = 11)	*P*
Age (years)	63.6 ± 6.0	66.9 ± 3.0	.188
Abnormal DRE, n (%)	9 (25.7%)	3 (27.3%)	.918
PSA (ng/mL), median	6.08 (1.1-40.0)	7 (2.5-19.1)	.511
PSAD (median)	0.17 (0.03-0.76)	0.12 (0.05-0.22)	.094
Prostate volume (median) (ml)	40 (13-120)	52.5 (28-131)	.021
Max tumor length of core (mm)	7.4 ± 4.2	5.4 ± 3.4	.179
Number of cores per index lesion	4.2 ± 1.4	4.2 ± 1.4	.232
Number of positive cores	7.1 ± 4.2	5.6 ± 4.2	.271
Index lesion diameter (mm)	15.0 ± 8.6	16.5 ± 12.0	.846

DRE, digital rectal examination; PSA, prostate-specific antigen; PSAD, prostate-specific antigen density.
